# Compensatory strategies below the behavioural surface in autism: a qualitative study

**DOI:** 10.1016/S2215-0366(19)30224-X

**Published:** 2019-09

**Authors:** Lucy Anne Livingston, Punit Shah, Francesca Happé

**Affiliations:** a Social, Genetic, and Developmental Psychiatry Centre, Institute of Psychiatry, Psychology, and Neuroscience, King's College London, London, UK; b Department of Psychology, University of Bath, Bath, UK

## Abstract

**Background:**

Little is known about the compensatory profile in autism; that is, people with autism spectrum disorder who show few symptoms in their behavioural presentation, despite continuing to report autism-related cognitive difficulties or differences. Even less is known about the specific compensatory strategies that these individuals use to disguise autism at the behavioural surface, both in the clinic and everyday life. It is also currently unclear whether individuals without a formal autism diagnosis, but experiencing autistic-like difficulties, use similar compensatory strategies, potentially enabling them to sit below the diagnostic threshold. This study aimed to investigate social compensatory strategies, and their effect on diagnosis and clinical outcome, in adults with and without autism.

**Methods:**

In this study, individuals aged 18 years or older who responded to a study advert that was distributed worldwide via social media and the UK National Autistic Society formed a convenience sample. Participants self-reported their use and experiences of compensatory strategies using an online platform. Novel analyses, including a qualitative thematic approach, were used to interpret their responses and gain insight into compensatory strategies in autism.

**Findings:**

Between Oct 19, 2017, and Jan 2, 2018, 136 adults (58 had a clinical diagnosis of autism, 19 self-identified but were not formally diagnosed as autistic, and 59 were not diagnosed or self-identified, but nevertheless reported social difficulties) completed the online study questions. The findings suggested that there are multiple compensatory strategies with distinct characteristics, individual and environmental factors that modulate compensatory strategy use and success, positive (social relationships, independence, employment) and negative (poor mental health, late diagnosis) outcomes associated with compensatory strategy use, and that individuals without a diagnosis use compensatory strategies that are qualitatively similar to individuals with a diagnosis.

**Interpretation:**

Increased awareness and measurement of compensatory strategy use in autism should guide future diagnostic guidelines, towards improved diagnostic accuracy and support for people with autism spectrum disorder whose cognitive difficulties are not immediately evident in observable behaviour.

**Funding:**

UK Medical Research Council and UK National Institute for Health Research.

## Introduction

Autism spectrum disorder is characterised by social communication impairments and by repetitive and restricted behaviours.^1^ Developmental trajectories in autism spectrum disorder are heterogeneous,^2^ but there is insufficient understanding of this phenomenon. In particular, it is unclear why some autistic people appear neurotypical in their behavioural presentation, despite having autism-related cognitive difficulties or differences (eg, in Theory of Mind [ToM]).^3^ One explanation is that individuals compensate for their cognitive difficulties by using alternative cognitive routes to achieve neurotypical behaviour.^4^ Such compensation has typically been investigated in the social domain (eg, compensation for ToM difficulties), although non-social cognitive difficulties might also be compensated for. Please note that we use both identity-first language (autistic person) and person-first language (person with autism) throughout to reflect variability in the language preferences of the autism community and the study participants.

Compensation might be an adaptive trajectory that can be differentiated from other trajectories in psychiatry, such as resilience,^5^, ^6^ in which a negative outcome is avoided, behaviourally, cognitively, and neurologically, despite exposure to risk. Instead, autistic compensators, despite apparent lack of observable autistic behaviour, continue being autistic at the neurocognitive level.^4^ Importantly, compensation can generate challenges in diagnosing and supporting these individuals.^7^ Because autism spectrum disorder is diagnosed by behaviour alone,^1^ compensators might not receive a diagnosis and support until later in life, if at all.^8^, ^9^ This issue is thought to be particularly acute in females, who are less likely than males to be diagnosed with autism spectrum disorder despite similar underlying autistic characteristics.^9^, ^10^, ^11^ Even for people with a diagnosis, a neurotypical appearance due to compensation might result in support needs being underestimated in educational and workplace settings. Additionally, compensation is thought to contribute to poor mental health in autism. Compensatory attempts are taxing, need to be sustained over time, and are often unsuccessful, resulting in a cost to wellbeing.^4^, ^9^, ^12^, ^13^

**Research in context**

**Evidence before this study**

We searched PubMed for articles published from database inception to March 31, 2018, with the broad terms (“compensat*” OR “camouflag*”) AND (“autism” OR “ASD”), given the limited literature on this topic. There were no language restrictions, and we did not specify an age range. This search was supplemented by reviewing reference lists and forward citations of relevant articles, with a focus on the reference list in a recent review on compensation in neurodevelopmental disorders. Across a small body of quantitative and qualitative studies generated, there was basic evidence for the phenomenon of compensation in autism—eg, by measuring the discrepancy between observable social behaviour and social cognitive task performance—and its cognitive correlates (higher intelligence quotient and executive function, but lower mental wellbeing). No study, however, had directly investigated compensatory strategies used by people with autism in social situations, and there were no qualitative analyses reported in previous research specifically on compensation.

**Added value of this study**

This first study, to our knowledge, to directly investigate compensatory strategies in autism, provided evidence for the existence of several compensatory strategies, and their modulation by various internal and external factors. We found both positive and negative consequences of the use of compensatory strategies. In particular, the study highlighted that compensatory strategies might be a barrier to a diagnosis of autism, and their use might have negative consequences on mental health and wellbeing.

**Implications of all the available evidence**

Improved awareness of compensation among clinicians will help them to detect compensatory strategies and support autistic compensators who might otherwise use (or misuse) these strategies at a cost to their mental wellbeing. In the long term, this study will feed into the refinement of diagnostic manuals that mention cursorily, but currently contain little guidance on, compensatory strategies in autism and co-occurring mental health conditions.

A small but fast-growing body of research has explored compensatory ability and the related phenomenon of camouflaging^14^ (see appendix p 1 for key definitions). In this research, compensation has been quantified as the discrepancy between perceived social abilities (observable behaviour) and actual underlying abilities (social cognitive task performance).^14^, ^15^ Other approaches have measured compensation or camouflaging through self-report^16^ and observation.^17^, ^18^ Across these studies, evidence suggests that compensation is associated with a higher intelligence quotient (IQ)^15^ and executive function,^14^, ^15^ which has been interpreted as intellectually conceived learned strategies. Data also indicate a link between compensation and anxiety,^15^, ^19^ depression,^14^ and suicidal ideation.^20^ This link might be due to socially motivated autistic people compensating without success, which reduces their self-esteem and mental wellbeing.^13^

Despite recent progress, this research has largely been theoretical or correlational, shedding little light on the strategies underlying compensation in autism spectrum disorder. There are several other gaps in knowledge that we aimed to address in this study. First, research has focused on shallow compensation, which reflects compensatory strategies (eg, mimicking others' gestures) that are inflexible, prone to breakdown, and therefore not effective in all contexts. Such strategies enable one to disguise, but not necessarily overcome, social cognitive difficulties. However, it is likely other, more sophisticated strategies involving deep compensation exist, such as detail-focused analysis of social information,^4^, ^21^ which might allow a person to solve ToM and have fairly flexible social understanding, albeit via an atypical route.^4^ Second, research has focused on people with a clinical diagnosis. However, since compensation promotes neurotypical behaviour, the most successful compensators—despite experiencing autistic cognitive difficulties—might sit below the diagnostic threshold for autism spectrum disorder.^4^ Finally, understanding the complex experiences of compensation of autistic people might be realisable only through qualitative methods. Overall, we suggest that compensation requires further investigation, to improve diagnostic processes and clinical support for people with autism. We aimed to investigate social compensatory strategies in adults with and without an autism diagnosis.

## Methods

### Study design, participants, and procedures

In this study, individuals who responded to a study advert (appendix p 2) formed a convenience sample. The advert was distributed worldwide via social media and the UK National Autistic Society, to be inclusive and gain as representative sample as possible. We aimed to give a very wide pool of individuals, irrespective of gender, country of residence, and diagnostic status, the opportunity to participate. Individuals were eligible to take part in the online study if they were aged 18 years or older, and the advert made clear that they did not require a formal autism diagnosis to participate. Participants with a clinical autism diagnosis were assigned to the diagnosed group, those who self-identified but were not formally diagnosed as autistic were assigned to the self-identified group, and those who were not diagnosed or self-identified, but nevertheless reported social difficulties were assigned to the non-diagnosed group. Participants were requested to provide information about their sex and gender, their diagnosis (if they had one) and details of the health-care professionals who made the diagnosis, any comorbid psychiatric diagnoses, their country of residence, employment status, and whether or not they lived independently. Participants self-reported autistic behaviours by completing the ten-item autism spectrum quotient^22^ and answered open-ended questions about their social compensatory strategies. Participants also reported how successful and tiring their strategies were, and the likelihood of recommending them to others with social difficulties (see appendix pp 2–4 for full details). To avoid fatigue, participants were not required to complete the study in one sitting and had two weeks to participate after beginning the study. Ethical approval for the study was obtained from King's College London Ethics Committee (Psychiatry, Nursing and Midwifery Research Ethics Subcommittee), and all participants gave informed consent.

### Data analysis

Participants who answered all questions in the online study were included in the analysis. There were three research questions. First, what are the compensatory strategies? Second, are compensatory strategies in diagnosed and non-diagnosed people qualitatively similar? Third, how do compensatory strategies affect diagnosis and clinical outcome? We assessed open-ended questionnaire responses using thematic analysis. We followed the six-step process by Braun and Clarke.^23^ More specifically, we used an inductive, rather than theoretical, approach to qualitatively analyse the data. Themes were identified at the semantic (ie, explicit), rather than latent (ie, interpretive) level. The entire dataset (ie, all participants' full responses) was coded by one author (LAL) using NVivo, version 11, to capture a rich description of the whole dataset, but paying particular attention to data patterns associated with the broad research questions. Data codes were collated to generate initial themes (ie, patterns within the dataset). Finally, themes were refined by re-examining the coherence of data codes within each theme and the validity of each theme in relation to the whole dataset.

All authors collectively agreed on wording of themes and the thematic map. Participants did not give their feedback on the results. Quotations supporting the themes and subthemes and a comprehensive list of strategies are shown in the appendix (pp 6–11). In addition to using Braun and Clarke's process to derive themes and subthemes, and in line with the flexible nature of their method, we computed theme and subtheme endorsement (ie, the frequency of participants who endorsed each theme and subtheme; appendix pp 12–13). Quantifying theme endorsement represents an extension to thematic analysis^24^ that was required to compare compensatory strategies and their outcomes between diagnosed, self-identified, and non-diagnosed groups.

The diagnosed, self-identified, and non-diagnosed groups were compared on theme endorsement using Fisher's exact tests and on successful, tiring, and recommendation ratings of compensation using Kruskal-Wallis tests. All quantitative analyses were computed using SPSS, version 24.

### Role of the funding source

The funders of this study had no role in study design, data collection, data analysis, or data interpretation. The corresponding author had full access to all the data in the study and had final responsibility for the decision to submit for publication.

## Results

Between Oct 19, 2017, and Jan 2, 2018, 136 participants completed open-ended questions on the online platform (table 1). The diagnosed group had 58 participants, the self-identified group had 19 participants, and the non-diagnosed group had 59 participants.

**Table 1:** Participant characteristics

		**Diagnosed group (n=58)**	**Self-identified group (n=19)**	**Non-diagnosed group (n= 59)**	**Comparisons**		
					p value	Effect size	Direction of effect
Age, years		35·8 (11·5; 18–70)	40·2 (11·1; 25–64)	33·9 (14·8; 18–77)	0·11	η^2^=0·03	..
Age at diagnosis, years		30·1 (13·8; 3–70)	..	..	..	..	..
Autism-spectrum quotient score*		8·0 (1·9; 1–10)	7·7 (1·9; 3–10)	4·9 (2·3; 1–10)	<0·001	η^2^=0·32	Diagnosed and self-identified > non-diagnosed
Number of words used in responses to open-ended questions		1362·2 (895·2; 174–4226)	1950·7 (1720·5; 221–6403)	1221·5 (743·8; 205–4191)	0·092	η^2^=0·04	..
Intelligence quotient†		4·7 (2·1; 0–7)	4·8 (1·9; 0–7)	4·7 (1·8; 1–7)	0·83	η^2^=<0·01	..
Gender		..	..	..	..	..	
	Female	37 (64%)	9 (47%)	51 (86%)	0·002	ϕ=0·36	Non-diagnosed > diagnosed and self-identified
	Male	13 (22%)	8 (42%)	8 (14%)	..	..	..
	Other‡	8 (14%)	2 (11%)	0	..	..	..
Comorbid diagnoses							
	Developmental disorders	9 (16%)	1 (5%)	2 (3%)	0·066	ϕ=0·21	..
	Anxiety disorders	21 (36%)	9 (47%)	18 (31%)	0·42	ϕ=0·12	..
	Obsessive-compulsive	4 (7%)	1 (5%)	2 (3%)	0·77	ϕ=0·07	..
	Depressive disorders	13 (22%)	5 (26%)	12 (20%)	0·85	ϕ=0·05	..
	Bipolar disorder	1 (2%)	0	1 (2%)	$0\cdot \bar{9}$	ϕ=0·05	..
	Eating disorders	1 (2%)	0	0	0·57	ϕ=0·10	..
	Personality disorders	1 (2%)	0	2 (3%)	$0\cdot \bar{9}$	ϕ=0·08	..
	Trauma or stress disorders	2 (3%)	1 (5%)	1 (2%)	0·65	ϕ=0·07	..
	Schizophrenic disorders	0	0	0	..	..	..
	Other	3 (5%)	1 (5%)	0	0·20	ϕ=0·15	..
Misdiagnoses							..
	Developmental disorders	6 (10%)	0	1 (2%)	0·075	ϕ=0·20	..
	Anxiety disorders	10 (17%)	3 (16%)	3 (5%)	0·12	ϕ=0·18	..
	Obsessive-compulsive	2 (3%)	0	0	0·44	ϕ=0·14	..
	Depressive disorders	7 (12%)	3 (16%)	4 (7%)	0·39	ϕ=0·11	..
	Bipolar disorder	2 (3%)	0	1 (2%)	0·76	ϕ=0·08	..
	Eating disorders	1 (2%)	2 (11%)	0	0·027	ϕ=0·24	Self-identified > non-diagnosed
	Personality disorders	3 (5%)	0	0	0·13	ϕ=0·17	..
	Trauma or stress disorders	1 (2%)	0	0	0·57	ϕ=0·10	..
	Schizophrenic disorders	3 (5%)	0	0	0·13	ϕ=0·17	..
	Other	2 (3%)	3 (16%)	0	0·006	ϕ=0·27	Self-identified > non-diagnosed
Residence							
	UK	39 (67%)	14 (74%)	50 (85%)	0·083	ϕ=0·19	..
	USA or Canada	8 (14%)	3 (16%)	5 (8%)	0·58	ϕ=0·09	..
	Europe	5 (9%)	1 (5%)	1 (2%)	0·18	ϕ=0·15	..
	Australasia	5 (9%)	1 (5%)	1 (2%)	0·18	ϕ=0·15	..
	Africa	1 (2%)	0	0	0·57	ϕ=0·10	..
	Asia	0	0	2 (3%)	0·63	ϕ=0·14	..
Employment status							
	Full time	23 (40%)	7 (37%)	12 (20%)	0·061	ϕ=0·20	..
	Part time	10 (17%)	2 (11%)	11 (19%)	0·78	ϕ=0·07	..
	Voluntary	1 (2%)	3 (16%)	3 (5%)	0·053	ϕ=0·21	..
	Student	13 (22%)	3 (16%)	28 (47%)	0·003	ϕ=0·29	Non-diagnosed > diagnosed and self-identified
	Unemployed	11 (19%)	4 (21%)	5 (8%)	0·19	ϕ=0·16	..
Live independently							
	Yes	41 (71%)	13 (68%)	43 (73%)	0·93	ϕ=0·03	..
	No	17 (29%)	6 (32%)	16 (27%)	..	..	..

Data are mean (SD; range) or n (%). Percentages might not sum to 100% because of rounding. One-way ANOVAs compared the groups on age, autism-spectrum quotient score, number of words, and intelligence quotient. Fisher's exact tests compared the groups on the categorical variables. η^2^=0·01 indicates a small effect size, η^2^=0·06 indicates a medium effect size, and η^2^=0·14 indicates a large effect size. ϕ=0·1 indicates a small effect size, ϕ=0·3 indicates a medium effect size, and ϕ=0·5 indicates a large effect size.

* Autism-spectrum quotient^22^ score (self-reported autistic behaviours), with a maximum score of 10.

† Intelligence quotient estimated using the 8-point scale of International Standard Classification of Education, with a maximum score of 7;^25^ higher scores reflect higher educational attainment.

‡ Included transgender males, transgender females, and non-binary individuals.

Compensation was reported as a secondary route (theme; figure) for social interaction because the primary route was unavailable. It enabled “passing” as neurotypical in certain situations through a cognitively taxing (subtheme; panel; table 2) process. Compensation involved using intellectual and executive functions to regulate social behaviour, such as intellectually conceived patterns about social norms (eg, making eye contact), preplanning social niceties (eg, asking others questions about themselves), and switching between social rules. Compensation was therefore more difficult when distracted or stressed, difficult to sustain, and resulted in mental fatigue. Additionally, compensatory strategies had an upper limit (subtheme) because they did not function in all situations or were too slow and inflexible in fast-moving social interaction. Overall, participants reported that the most difficult aspects of social interaction, such as responding to unexpected turns of conversation, could not be achieved via the secondary route of compensation.

**Figure:**
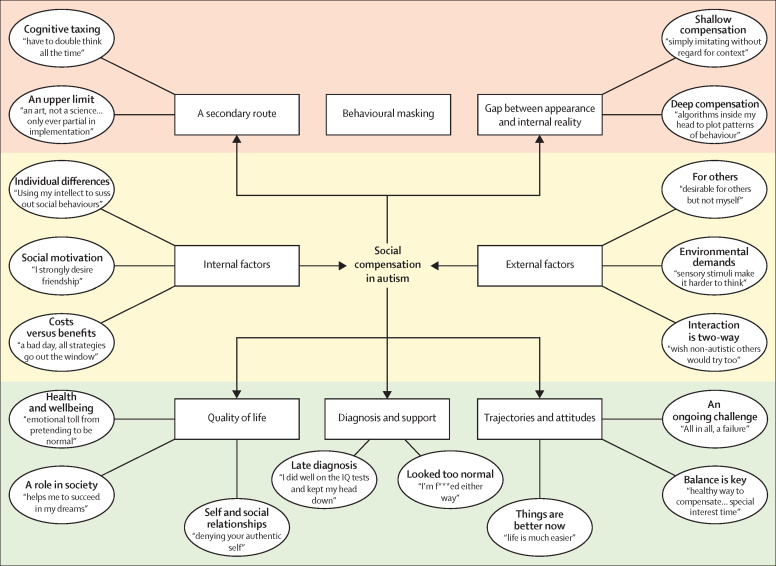
Thematic map of the eight themes (rectangular) and 18 subthemes (oval) in social compensation in autism Each subtheme is accompanied by an example quotation from the dataset. A list of quotations and compensatory strategies are provided in the appendix (pp 6–11).

Panel **Quotations supporting themes and subthemes**

**Cognitively taxing**

“Constant overthinking of possibilities of what to say, how it will come across, what others are and are not saying, the connotations of every word, sentence structure, emphasis, body language, as well as all of the above combined in a giant matrix of thought!”

*Male, non-diagnosed, aged 25–30 years*

“It is always a make-do, perhaps like a foreign language. Even though one might have adopted it to a good extent, it's never native.”

*Male, non-diagnosed, aged 31–40 years*

**An upper limit**

“I think I could make ‘all the right choices’ in social situations if I could choose offline with more time to reflect and from afar, but real situations are far trickier.”

*Male, self-identified, aged 25–30 years*

**Gap between appearance and internal reality**

“It's what going on cognitively, not behaviourally and people don't see that. It's frustrating because I don't…get the support or understanding that I need.”

*Female, non-diagnosed, aged 25–30 years*

“We have a hell of a lot of difficulties and just because we hide them doesn't mean they don't exist.”

*Female, diagnosed, aged 41–50 years*

**Shallow compensation**

“There are obvious flaws, if you are observant – I repeat myself or use tv/film phrases and sometimes say things which are out of place.”

*Female, diagnosed, aged 41–50 years*

“These ‘unspoken’ rules do not always apply to all people and all contexts. You have to re-evaluate the situation or even the same person all the time.”

*Female, non-diagnosed, aged 25–30 years*

**Deep compensation**

“I think I observe patterns in behaviour and then try to transfer this. So if a person is behaving x/y/z types of ways, they could be feeling or thinking what so and so people had felt. It's almost a case of systematically storing little patterns in each person and the context, so I can refer to it in future.”

*Female, diagnosed, aged 25–30 years*

**Behavioural masking**

“It's usually easier to be just like everybody else and not stand out. Often it's just easier to be another brick in the wall.”

*Female, non-diagnosed, aged 25–30 years*

**Individual differences**

“Adaptability…my bad-ass superpower. I've learned to survive no matter what.”

*Female, self-identified, aged 51–60 years*

**Social motivation**

“I strongly desire friendship, but am aware that I am not very good at initiating it and even worse at maintaining it…despite my awareness, my ability to counteract my poor social skills lags behind. In short, now I know that I am the problem, but I still don't know how to fix myself very well.”

*Female, diagnosed, aged 31–40 years*

**Costs versus benefits**

“If I am having a bad day, all strategies go out the window, socialisation is no longer a priority, I just need to be alone.”

*Female, diagnosed, aged 18–24 years*

**For others**

“Compensation is born from necessity. We have extensive experience of how cruel people are.”

*Male, self-identified, aged 41–50 years*

“I was simply fed up with having very few friends, being disliked and ostracised by my peers and being bullied…I finally snapped at around 16…I didn't want to screw up this time.”

*Female, self-identified, aged 25–30 years*

**Environmental demands**

“Sensory environment. It can wipe out 100% of my ‘coping energy’ in moments.”

*Female, diagnosed, aged 51–60 years*

“I also perceive myself as more skilled in adulthood because I have spent most of my adult life in the UK. Rules and social norms are different here…a much easier fit for many people on the spectrum, because some of the things that come more naturally to us are valued in British culture—a certain amount of reserve, reticence, not treating everyone you meet like they are instantly a friend.”

*Female, diagnosed, aged 25–30 years*

**Interaction is two-way**

“[I] give informed opinions about some issue of interest to my interlocutor in place of small talk…I am stuck when I meet people who have no interests and extreme extroverts.”

*Female, diagnosed, aged 41–50 years*

“With autistic people, who speak my language…it goes fantastically well most of the time. Or with some non-autistic people who are very comfortable without a lot of eye contact and social irrelevances, so don't mind me being me. Any time I'm having to pretend, it's exhausting, inauthentic, and ultimately pointless.”

*Female, diagnosed, aged 51–60 years*

**Late diagnosis**

“The big problem arose when my peers moved on to applying for jobs and being in the real world. I knew that I could never function, as an office boy, let alone a management consultant.”

*Male, self-identified, aged 41–50 years*

“We assume a very normal aspect when seeing our GP…video recording standard behaviour is the only way that a GP could actually witness what other people see.”

*Male, self-identified, aged 41–50 years*

**Looked too normal**

“I think I'm f***ed either way…because people think that I can take more of their s*** if I compensate than I actually can.”

*Female, diagnosed, aged 31–40 years*

“A lot of people who know me superficially express surprise that I am autistic. I don't take it as a compliment and I often want to respond with ‘Do you realise how much damn hard work it is to seem this normal?’”

*Transgender female, diagnosed, aged 41–50 years*

**Health and wellbeing**

“I have planned three methods for my own suicide…I have lost great people in my life and destroyed previous careers and relationships. All of this, I put down to compensating.”

*Male, diagnosed, aged 25–30 years*

“The worst aspect of my compensation techniques is that they work on the basis that I am not good in social settings and so by acting out my compensation techniques I reinforce this idea that I am bad at socialising and [this] lowers my confidence.”

*Male, non-diagnosed, aged 18–24 years*

**A role in society**

“It cuts down the pain and makes me employable. …To not compensate would make life more unhappy for me and those with whom I force to interact with me.”

*Male, self-identified, aged 41–50 years*

“I have little energy left at the end of the workday, I can't keep up with the cleaning of my house or feed myself. It's hard to imagine myself as a mother…it's not because I'm not competent.”

*Female, diagnosed, aged 31–40 years*

**Self and social relationships**

“The inability to mask or compensate beyond the initial stages of a relationship has meant I have never developed the social capital which all people need to succeed.”

*Male, diagnosed, aged 31–40 years*

“I feel like I am acting most of the time and when people say that I have a characteristic, I feel like a fraud because I've made that characteristic appear.”

*Female, non-diagnosed, aged 41–50 years*

**Things are better now**

“With compensation, I have a job in which people respect my work and ask for my help and opinions…I am liked by my colleagues and friends…I haven't lived on the edge, lost and lonely, as I could have. I have been super super lucky.”

*Female, non-diagnosed, aged 31–40 years*

**Balance is key**

“I am more honest now with myself and others and I limit my interactions to keep myself mentally and, therefore, physically healthy. I have more energy for twisting the world because I am twisting less of it.”

*Male, diagnosed, aged 25–30 years*

**An ongoing challenge**

“I still have no clues about what can I do to make it better. I'm more alone than ever.”

*Male, self-identified, aged 41–50 years*

**Table 2:** Number of participants endorsing each theme or subtheme, by gender, group, and autistic behaviours

		Gender*		**Group**			**Autistic behaviours**†	
		Male (n=29)	Female (n=97)	Diagnosed (n=58)	Self-identified (n=19)	Non-diagnosed (n=59)	High (n=89)	Low (n=47)
A secondary route								
	Cognitively taxing	26 (90%)	92 (95%)	55 (95%)	18 (95%)	55 (93%)	83 (93%)	45 (96%)
	An upper limit	22 (76%)	59 (61%)	40 (69%)	13 (68%)	35 (59%)	61 (69%)	27 (57%)
Gap between appearance and internal reality								
	Shallow compensation	26 (90%)	93 (96%)	55 (95%)	17 (89%)	57 (97%)	88 (99%)‡	41 (87%)‡
	Deep compensation	27 (93%)	96 (99%)	57 (98%)	19 (100%)	57 (97%)	88 (99%)	45 (96%)
Behavioural masking		29 (100%)	96 (99%)	57 (98%)	19 (100%)	59 (100%)	88 (99%)	47 (100%)
Internal factors								
	Individual differences	29 (100%)	96 (99%)	57 (98%)	19 (100%)	59 (100%)	88 (99%)	47 (100%)
	Social motivation	24 (83%)	91 (94%)	50 (86%)	19 (100%)	56 (95%)	81 (91%)	44 (94%)
	Costs versus benefits	20 (69%)	75 (77%)	38 (66%)	16 (84%)	46 (78%)	64 (72%)	36 (77%)
External factors								
	For others	26 (90%)	95 (98%)	56 (97%)	17 (89%)	58 (98%)	88 (99%)§	43 (91%)§
	Environmental demands	28 (97%)	90 (93%)	55 (95%)	18 (95%)	55 (93%)	85 (96%)	43 (91%)
	Interaction is two-way	27 (93%)	96 (99%)	56 (97%)	18 (95%)	59 (100%)	88 (99%)	45 (96%)
Diagnosis and support								
	Late diagnosis	24 (83%)	87 (90%)	52 (90%)	18 (95%)	50 (85%)	83 (93%)§	37 (79%)§
	Looked too normal	21 (72%)	77 (79%)	47 (81%)	15 (79%)	43 (73%)	70 (79%)	35 (74%)
Quality of life								
	Health and wellbeing	27 (93%)	96 (99%)	55 (95%)	18 (95%)	59 (100%)	86 (97%)	46 (98%)
	A role in society	17 (59%)	62 (64%)	40 (69%)	11 (58%)	37 (63%)	56 (63%)	32 (68%)
	Self and social relationships	25 (86%)	83 (86%)	48 (83%)	16 (84%)	53 (90%)	78 (88%)	39 (83%)
Trajectories and attitudes								
	Things are better now	19 (66%)	67 (69%)	34 (59%)§	13 (68%)§	47 (80%)§	57 (64%)	37 (79%)
	Balance is key	24 (83%)	86 (89%)	50 (86%)	18 (95%)	50 (85%)	79 (89%)	39 (83%)
	An ongoing challenge	16 (55%)	39 (40%)	23 (40%)	9 (47%)	27 (46%)	42 (47%)	17 (36%)

Data are number (%) of participants giving at least one example fitting each theme or subtheme. Each participant received a maximum of one count per theme or subtheme. Fisher's exact tests compared frequencies across group, gender, and autistic behaviours. Full p values and effect sizes are in the appendix (pp 12–13).

* Participants reporting other gender were not included in these analyses.

† Collapsing across diagnostic groups, participants were split into high and low autism-spectrum quotient score groups: the high group had a score of 6 or more, in line with the clinically significant cutoff,^22^ and the low group had a score of less than 6.

‡ p<0·01.

§ p<0·05.

Participants reported that compensation generated a gap between appearance and internal reality (theme), whereby they experienced social cognitive difficulties that went unnoticed by others. This gap partly stemmed from differing levels of compensatory strategy (see appendix pp 9–11 for list of strategies). Many strategies, involving shallow compensation (subtheme), were simple and inflexible (eg, laughing after joke cues). These strategies transferred poorly to new contexts and seldom reduced participants' social cognitive difficulties. Accordingly, this subtheme was endorsed more often by participants who self-reported more autistic behaviours (as determined by the autism-spectrum quotient score; table 2). Participants perceived that neurotypical individuals could often “see through” these strategies, which were also less effective when stressed and meeting new people. Deep compensation (subtheme), involving complex and flexible strategies, contributed to some improvements in social cognition. Some participants reported using pattern detection and internal data modelling (gesture + facial expression + context = particular mental state) to understand others. These strategies, although hard to implement at first, could become “second nature” with time.

Compensation was distinguishable from behavioural masking (theme). Whereas compensation generated new social behaviours, masking regulated existing behaviours, such as decreasing social behaviours thought by society to be undesirable (eg, talking too much) and increasing behaviours thought to be desirable (eg, smiling). Masking strategies were simple and often automatic, and allowed blending into the background, but were less effective in supporting social interaction. Masking was considered less autism-specific than compensation, given that neurotypical people show masking when required (eg, hiding controversial opinions).

A range of internal factors (theme) were found to drive compensation and modulate strategy success. Compensation was linked to individual differences (subtheme) in autism-related (eg, detail focus) and non-autism-related (eg, intelligence) processes, to plan, execute, and refine strategies. Persisting with compensation, despite failed attempts, was also a key internal factor underpinning the use of compensatory strategies. Many participants reported social motivation (subtheme) driving them to compensate to develop meaningful relationships. This motivation was evident from descriptions of reputational concerns (avoiding appearing socially inept), concern for others (avoiding hurting feelings), and distress from any social rejection following compensatory efforts. To modulate compensatory efforts, many participants described compensating after logically assessing the costs versus benefits (subtheme). For example, compensation was considered worthwhile to make a positive impression towards a friendship, but not for interactions with inconsequential strangers. In superficial interactions, masking was preferred over compensatory strategies to conserve resources.

Compensation, and its success, was also described in relation to several external factors (theme). Compensation was for others (subtheme). It was necessary to avoid rejection and ostracism, and often stemmed from bullying or pressure to conform from a young age. Nevertheless, fitting neurotypical peoples' interaction style (eg, eye-contact or small talk) was viewed as vital for achieving life goals (eg, independence and employment). These external pressures to compensate were greater in participants who self-reported more autistic behaviours. More generally, these pressures were greater when meeting new people, particularly those with good social skills, than with family and friends, and changed over the lifespan, being higher in adolescence and lower in older age.

Environmental demands (subtheme), such as social interactions in loud and bright rooms, made it difficult for participants to use compensatory strategies. Additionally, group situations, involving multiple social cues and unstructured social settings (eg, parties), were more demanding on compensatory resources than one-to-one structured interaction (eg, doctor's appointment). Therefore, many individuals reported “passing” as neurotypical in environments with low demands, but appearing socially atypical in those with higher demands. Environmental demands also changed over time and in certain societies and cultures that were intolerant of atypical behaviour. Similarly, because interaction is two-way (subtheme), compensation success was linked to the interaction partner. Participants reported that it was difficult to compensate when others had social difficulties, because there were greater demands on their mental resources. Conversely, similarities between the compensator and their interaction partner (eg, special interests) reduced the need to compensate. Indeed, many participants reported a better understanding of atypical versus neurotypical minds, which supported social interaction regardless of compensation.

Compensation had a profound impact on autism diagnosis and support. Most of the diagnosed group received a late diagnosis (subtheme) in adulthood, and compensation helped to explain why autism was missed in childhood or adolescence. More broadly, individuals who self-reported more autistic behaviours (regardless of diagnosis), endorsed this subtheme more than individuals with fewer autistic behaviours. Despite individuals having always felt different, compensatory strategies supported success (eg, academically) in earlier life; hence, autistic characteristics were overlooked by parents and teachers. Additionally, some participants' autistic characteristics were not sufficiently impairing to warrant diagnosis until adulthood. Participants described examples of accommodation during childhood (eg, parent communicating on their behalf), which had enabled them to compensate sufficiently. For most participants, when demands (eg, living independently) increased in adulthood, their compensatory strategies became insufficient or their autistic characteristics impaired their daily functioning. In some cases, life-changing events (eg, death of a partner) led to such compensation breakdown. Even after recognising autism-related difficulties, or compensation breakdown, many participants reported that undergoing an autism assessment was challenging in adulthood. Participants attributed this difficulty to clinicians' lack of awareness about compensation, sometimes resulting in misdiagnoses before a diagnosis of autism spectrum disorder (table 1).

Compensation typically resulted in a lack of support in adulthood because participants looked “too normal” (subtheme). Employed participants reported that employers and colleagues held them to a neurotypical standard, which resulted in social errors not being interpreted in the context of autism. Because autistic characteristics went undetected by others, many participants reported that it was difficult to request, and they were unlikely to receive, workplace accommodations. Furthermore, many participants reported that, following disclosure of their autistic characteristics or diagnosis, they were disbelieved and poorly supported because of their neurotypical presentation. This lack of support meant that exhaustion and burnout was frequent.

Compensation was linked to participants' quality of life (theme), particularly in relation to health and wellbeing (subtheme). A minority of participants felt that compensation was either unrelated to or improved their wellbeing. However, for many participants, anxiety and self-consciousness in social situations drove compensation, and compensation was itself reported as stressful and exhausting. Many compensators associated their strategies with experiencing anxiety, depression, and suicidal ideation (table 1). This association was attributed to isolation, low self-esteem, and rumination about social failings when compensatory efforts were unsuccessful. Some participants also linked compensation with poor physical health (eg, needing more sleep or feeling nauseous).

Despite potential negative consequences, compensation was still considered to be important for increasing life opportunities, and thereby having a role in society (subtheme). Compensation enabled individuals to perform daily tasks that involved communicating with others (eg, accessing services) and to seek employment. Some participants, however, stressed that although compensatory strategies facilitated gaining employment (eg, in interviews), they were not always sufficient to maintain employment and switching jobs was often necessary. Additionally, cognitive demands of using compensatory strategies throughout the working day were reported to affect participants' ability to perform daily living tasks, so they incurred personal costs while pursuing a role in society.

Participants reported positive and negative consequences of compensation for their sense of self and social relationships (subtheme). Compensation helped to foster confidence and increased feelings of connectedness to others. Many participants, however, noted that compensation was not always sufficient because their strategies were unable to convert acquaintances into friendships, or their differences were “found out”, with negative consequences for social relations. Similarly, some participants reported that being socially motivated, without adequate strategies, led them to social efforts that were not reciprocated. Additionally, as compensation often involved some deception (eg, faking interests), some relationships were not based on a genuine connection and were therefore unsatisfying or unsustainable. This inadequate compensation often had an impact on individuals' attitudes towards themselves. Participants reported that compensation—widely described as “putting on a performance”—resulted in a diminished and uncertain sense of self.

There was great variability in trajectories and attitudes (theme; table 3) towards compensation over the lifespan. Some participants reported that compensatory strategies and quality of life improved from childhood to adulthood. These individuals now found social situations easier than earlier in life, through refining strategies, pursuing environments that supported strategy success, or improvements in social cognition (ie, deep compensation) with age. Overall, these individuals, who expressed that things are better now (subtheme), reported compensation as a positive worthwhile process. Other individuals highlighted that compensation had benefits, but respite from compensating was important: that is, balance is key (subtheme). These participants were less concerned with appearing neurotypical, often casting their autistic differences as strengths. They reported seeking environments that were accommodating of their autistic characteristics, such as workplaces in which non-social abilities were more important than social skills. This trajectory, characterised by a tendency to compensate less with age, was linked to improved mental and physical health. However, some individuals, who viewed compensation and its outcomes as an ongoing challenge (subtheme), reported that compensatory strategies were largely unsuccessful. Their life goals were unmet and they desired better strategies. These individuals were distinct from the balance is key trajectory in their attitudes towards compensation. They were more self-critical of their autistic characteristics and compensatory failures, and reported a negative impact of compensation on their wellbeing.

**Table 3:** Participant's attitudes towards social compensation

	**Total sample (n=136)**	Diagnosed group*(n=58)	**Self-identified group (n=19)**	**Non-diagnosed group (n=59)**
**How successful?**				
Extremely successful	52 (38%)	18 (31%)	6 (32%)	28 (47%)
Somewhat successful	76 (56%)	34 (59%)	12 (63%)	30 (51%)
Neither	5 (4%)	3 (5%)	1 (5%)	1 (2%)
Somewhat unsuccessful	2 (1%)	2 (3%)	0	0
Extremely unsuccessful	1 (1%)	1 (2%)	0	0
**How tiring?**				
Extremely tiring	16 (12%)	9 (16%)	4 (21%)	3 (5%)
Somewhat tiring	48 (36%)	23 (40%)	11 (58%)	14 (24%)
Neither	30 (22%)	10 (17%)	4 (21%)	16 (27%)
Somewhat energising	27 (20%)	13 (23%)	0	14 (24%)
Extremely energising	14 (10%)	2 (3%)	0	12 (20%)
**Likely to recommend?**				
Definitely recommend	27 (20%)	12 (21%)	3 (16%)	12 (20%)
Likely to recommend	49 (36%)	17 (29%)	6 (32%)	26 (44%)
Neither	37 (27%)	14 (24%)	6 (32%)	17 (29%)
Unlikely to recommend	19 (14%)	11 (19%)	4 (21%)	4 (7%)
Definitely not recommend	3 (2%)	3 (5%)	0	0

Data are n (%). Participants rated how successful and tiring their strategies were, and the likelihood of recommending them to others with social difficulties. Kruskal-Wallis tests compared the three groups on successful, tiring, and recommendation ratings. No significant differences were found for successful (p=0·073) or recommendation (p=0·20) ratings. Significant differences were found for tiring ratings, χ^2^ (2)=20·85, p<0·001. Follow-up Mann-Whitney *U* tests found that the diagnosed and self-identified groups did not significantly differ (*U*=391·50, p=0·057, Bonferroni-corrected p=0·17). However, the self-identified (*U*=216·00, p<0·001, Bonferroni-corrected p<0·001) and diagnosed (*U*=1119·50, p=0·001, Bonferroni-corrected p=0·004) groups reported higher tiring ratings than the non-diagnosed group.

* One participant from the diagnosed group had missing data for the tiring and recommendation ratings.

## Discussion

We identified several compensatory strategies used by people with and without autism. Shallow strategies were in line with theoretical predictions of surface-level compensation in autism.^4^ These strategies represent a finite resource, involving non-social instead of social cognitive processes in social situations. They were more common in participants who reported more autistic behaviours and were linked to the negative consequences of compensation. This finding is consistent with findings that IQ and executive function, but also mental health difficulties, are positively correlated with compensation in people with autism spectrum disorder,^14^, ^15^ and reflects notions of executive function being a finite and depletable resource.^26^ Our findings also provide empirical support for the theorised distinction between shallow and deep compensation.^4^ Recent neuroimaging data suggest that neural markers of compensation, probably involving deep compensation, are found in some autistic people.^21^ Deep, compared with shallow, compensatory strategies seem to be linked to better outcomes in autism. A precise understanding of the causes, mechanisms, and consequences of shallow and deep compensation is yet to be established, and this study provides the impetus for such research. Additionally, in line with recent research,^12^, ^16^ our findings confirmed that compensation is different from masking in autism spectrum disorder. The now clearer qualitative distinctions between these phenomena will benefit future research into their potentially distinctive (neuro)cognitive underpinnings and differential consequences for autistic behaviour and clinical presentation.

We found that there are wide-ranging motivations and consequences of compensatory strategies. Notably, levels of social motivation were high, suggesting that autistic compensators might form a subgroup of people with autism who want relationships because they are socially rewarding. This notion fits with recent proposals that social motivation is not universally impaired in autism.^13^, ^27^ Equally, however, some participants reported using compensatory strategies irrespective of social–emotional rewards and for instrumental reasons. Following cost–benefit analyses, these participants reported a rational approach to compensating, which is consistent with more rational and less emotionally driven cognition in autism.^28^ Together, our results provide important clues about psychological constructs related to compensation to be investigated in future. Through such research, it is possible that autistic people might ultimately be supported with compensatory strategies that are aligned (or misaligned) with their motivations, which might increase positive and decrease negative consequences of compensation that have also been identified in this study.

This study highlights that compensation is modulated by several external societal pressures. This finding is in line with research that people with autism are, despite the negative impact on their wellbeing, driven to meet neurotypical society's expectations of behaviour.^19^ To address this issue, it has been suggested that (neurotypical) society could do more to accommodate autistic people,^29^ which we speculate might reduce the need for compensation in autism spectrum disorder. Specifically, neurotypical individuals could engage in compensatory efforts, perhaps by reducing their reliance on social niceties, to improve interactions with autistic people. This concept of two-way compensation warrants further research.

More broadly, our findings indicate that external environments modulate compensation given that people with autism have sensory difficulties in certain environments.^30^ Researchers and clinicians should account for the immediate environment (eg, sensory features of testing rooms) when measuring compensation and diagnostic features in autistic people. If the environment is modified appropriately, compensatory efforts, and resulting interactions, could be improved. Equally, however, compensation might lead to individuals appearing neurotypical in certain contexts (eg, one-to-one clinical assessment in a dimly lit room), yet socially atypical in others (eg, noisy open-plan offices). This finding is important and merits further consideration in research, when designing autism-friendly spaces, and during clinical assessments.

Compensation has other consequences for clinical outcomes and practice. The use of compensatory strategies, in agreement with previous research,^9^, ^12^, ^14^, ^15^, ^16^, ^19^, ^20^ was linked with poor mental health. Clinicians should therefore be aware that conditions co-occurring with autism might, in part, be related to compensation. In addition, having autism-related difficulties overlooked and misinterpreted by clinicians as a result of compensation represents a barrier to diagnosis of autism spectrum disorder. Recent evidence^31^ suggests that only 40% of UK general practitioners—the first point of contact for individuals seeking diagnosis—are confident in identifying autism spectrum disorder. Unsurprisingly, then, misdiagnoses and late diagnoses were frequently reported in our sample of compensators. Many individuals self-reporting high levels of autistic behaviours mentioned experiences relevant to a late diagnosis, whether they had been diagnosed or not. Although DSM-5^1^ acknowledges that autistic symptoms “may be masked by learned strategies”, no guidelines exist for detecting these strategies or whether they should be encouraged. Given the individual differences found in this study, we tentatively suggest that clinicians take an individualised approach when assessing and discussing compensatory strategies with people with autism. It is hoped that the current findings build awareness of compensatory strategies, towards better diagnostics and support for autistic people, and co-occurring conditions that might arise from compensation.^7^

Despite clinical implications, our findings suggest that previous research^12^, ^13^, ^14^, ^15^, ^16^, ^17^, ^18^, ^19^, ^20^ might have over-emphasised the negative aspects of compensation. Many participants reported that compensation was fundamental to fulfilling life experiences and deemed their strategies successful. The so-called best outcomes were reported by individuals who balanced compensatory strategy use with seeking environments compatible with their characteristics. It will be important to establish which compensatory strategies are most beneficial, and how their success might be maximised with changes to external environments irrespective of clinical intervention.

We report that non-diagnosed adults who experience social difficulties use compensatory strategies that are qualitatively similar to those of people with autism. Some of these individuals might meet autism spectrum disorder criteria, and our data indicate why they might not have sought a diagnosis. They reported compensation as less tiring than did the other groups and were more likely to endorse the things are better now subtheme. This finding is in line with suggestions that individuals who miss the diagnostic threshold for autism spectrum disorder might be superior compensators to diagnosed individuals.^4^ However, this study does not address whether non-diagnosed participants had social difficulties unrelated to autism; therefore, a detailed comparison of diagnosed versus non-diagnosed compensators will be required in future.

A limitation of our study was the high proportion of female, late-diagnosed, and well educated participants, thereby reducing generalisability of our findings to non-females, individuals diagnosed in childhood, and those with lower intellectual ability. This study will also not have captured subconscious compensatory processes because of self-report methodology. Future research will benefit from measuring self-reported (conscious) and neurocognitive (subconscious) markers of compensation in light of the strategies uncovered in the current study. More broadly, samples representative of the population, including early-diagnosed and late-diagnosed autistic people, and quantitative analyses, will be required. Such analyses will help to address outstanding questions about frequencies and sex differences in the use of compensatory strategies, and the causes and consequences of compensation for the diagnosis of autism spectrum disorder.

Overall, analysis of rich data in a large, heterogeneous sample generated novel insights into compensatory strategies in autism missing from previous research. It is hoped that this study will prompt further discussion around and consideration of compensation in autism, in both clinical and research settings, towards improved diagnostic accuracy and support for autistic people whose difficulties are not always evident at the behavioural surface.

## Data sharing

Raw qualitative data cannot be shared due to ethical restrictions. Anonymised quantitative data will be shared on reasonable request to the corresponding author.

## References

[1] American Psychiatric Association (2013). Diagnostic and statistical manual of mental disorders.

[2] Howlin P, Magiati I (2017). Autism spectrum disorder: outcomes in adulthood. Curr Opin Psychiatry.

[3] Livingston LA, Carr B, Shah P (2019). Recent advances and new directions in measuring theory of mind in autistic adults. J Autism Dev Disord.

[4] Livingston LA, Happé F (2017). Conceptualising compensation in neurodevelopmental disorders: reflections from autism spectrum disorder. Neurosci Biobehav Rev.

[5] Rutter M (2013). Annual research review: resilience—clinical implications. J Child Psychol Psychiatry.

[6] Lai M-C, Szatmari P (2019). Resilience in autism: research and practice prospects. Autism.

[7] McPartland JC (2019). Commentary: Autism's existential crisis: a reflection on Livingston et al. (2018). J Child Psychol Psychiatry.

[8] Lai M-C, Baron-Cohen S (2015). Identifying the lost generation of adults with autism spectrum conditions. Lancet Psychiatry.

[9] Bargiela S, Steward R, Mandy W (2016). The experiences of late-diagnosed women with autism spectrum conditions: an investigation of the female autism phenotype. J Autism Dev Disord.

[10] Ratto AB, Kenworthy L, Yerys BE (2018). What about the girls? Sex-based differences in autistic traits and adaptive skills. J Autism Dev Disord.

[11] Lehnhardt FG, Falter CM, Gawronski A (2016). Sex-related cognitive profile in autism spectrum disorders diagnosed late in life: implications for the female autistic phenotype. J Autism Dev Disord.

[12] Hull L, Petrides KV, Allison C (2017). “Putting on my best normal”: social camouflaging in adults with autism spectrum conditions. J Autism Dev Disord.

[13] Livingston LA, Shah P, Happé, F. Compensation in autism is not consistent with social motivation theory. *Behav Brain Sci* (in press).

[14] Lai M-C, Lombardo MV, Ruigrok ANV (2017). Quantifying and exploring camouflaging in men and women with autism. Autism.

[15] Livingston LA, Colvert E, Bolton P, Happé F, Social Relationships Study Team (2019). Good social skills despite poor theory of mind: exploring compensation in autism spectrum disorder. J Child Psychol Psychiatry.

[16] Hull L, Mandy W, Lai M-C (2019). Development and validation of the Camouflaging Autistic Traits Questionnaires (CAT-Q). J Autism Dev Disord.

[17] Dean M, Harwood R, Kasari C (2017). The art of camouflage: Gender differences in the social behaviors of girls and boys with autism spectrum disorder. Autism.

[18] Parish-Morris J, Lieberman MY, Cieri C (2017). Linguistic camouflage in girls with autism spectrum disorder. Mol Autism.

[19] Cage E, Troxell-Whitman Z (2019). Understanding the reasons, contexts and costs of camouflaging for autistic adults. J Autism Dev Disord.

[20] Cassidy S, Bradley L, Shaw R, Baron-Cohen S (2018). Risk markers for suicidality in autistic adults. Mol Autism.

[21] Lai M-C, Lombardo MV, Chakrabarti B (2019). Neural self-representation in autistic women and association with ‘compensatory camouflaging’. Autism.

[22] Allison C, Auyeung B, Baron-Cohen S (2012). Toward brief “red flags” for autism screening: The Short Autism Spectrum Quotient and the Short Quantitative Checklist for Autism in Toddlers in 1,000 cases and 3,000 controls. J Am Acad Child Adolesc Psychiatry.

[23] Braun V, Clarke V (2006). Using thematic analysis in psychology. Qual Res Psychol.

[24] Ryan GW, Bernard HR, Densin NK, Lincoln YS (2000). Data management and analysis methods. Handbook of qualitative research.

[25] UNESCO Institute for Statistics (2012). International Standard Classification of Education ISCED 2011.

[26] Powell LJ, Carey S (2017). Executive depletion in children and its impact on theory of mind. Cognition.

[27] Jaswal VK, Akhtar N (2018). Being vs. appearing socially uninterested: challenging assumptions about social motivation in autism. Behav Brain Sci.

[28] Shah P, Catmur C, Bird G (2016). Emotional decision-making in autism spectrum disorder: the roles of interoception and alexithymia. Mol Autism.

[29] Milton DEM (2012). On the ontological status of autism: the ‘double empathy problem’. Disabil Soc.

[30] Fletcher-Watson S, Happé F (2019). Autism: a new introduction to psychological theory and current debate.

[31] Unigwe S, Buckley C, Crane L, Kenny L, Remington A, Pellicano E (2017). GPs' confidence in caring for their patients on the autism spectrum: An online self-report study. Br J Gen Pract.

