# Supplementary material for: Compensatory strategies below the behavioural surface in autism: a qualitative study
Source: Lancet Psychiatry. 2019 Sep;6(9):766–77. doi: 10.1016/S2215-0366(19)30224-X (PMC6706698; doi:10.1016/S2215-0366(19)30224-X)
Supplement: Supplementary appendix [file mmc1.pdf]

THE LANCET
Psychiatry

Supplementary appendix

This appendix formed part of the original submission and has been peer reviewed. We post it as supplied by the authors.

Supplement to: Livingston LA, Shah P, Happé F. Compensatory strategies below the behavioural surface in autism: a qualitative study. *Lancet Psychiatry* 2019; published online July 23. http://dx.doi.org/10.1016/S2215-0366(19)30224-X.

Appendix

Supplementary Introduction

Key Definitions

Compensation – reflects the process(es) that enable an individual with a neurodevelopmental condition (e.g., autism) to show few symptoms in behaviour, despite persistence in underlying cognitive difficulties/differences. For example, an autistic person may 'compensate' for social cognitive difficulties, demonstrating neurotypical (non-autistic) social skills in behaviour, despite continuing to show difficulties on social cognitive tasks. This is proposed to be possible by using alternative neurocognitive routes to achieve the same behavioural output as neurotypical people. Compensatory processes may in part be unconscious, but also include deliberately employed strategies in conscious awareness. Supplementary Table 1 gives further details about the distinctions between different theorised forms of compensation (deep and shallow), adapted from a recent review paper on the topic.[1]

Compensatory Strategies – deliberately employed strategies to 'overcome' or 'bypass' the cognitive difficulty/difference in question, although some may become more 'second nature' with time. Compensatory strategies are likely to rely on cognitive (and underpinning neural) processes not typically used by neurotypical people. For example, an autistic person may use intellectual functions to 'work out' social rules (e.g., make eye contact with others) that otherwise come effortlessly to a neurotypical person via typical social cognitive routes.

Camouflaging – a phenomenon related to compensation in the literature,[2,3] which describes autistic people's efforts to hide their condition and 'blend in' to their neurotypical surroundings. It has been proposed to explain why autistic females may go undiagnosed despite equivalent autistic traits to males and is largely described at a behavioural rather than neurocognitive level of explanation, e.g., autistic people deliberately regulating their behaviour. Compensation may be a particular mechanism that falls under the broader phenomenon of 'camouflaging'.[3] Both concepts are fairly novel in the field and will necessarily undergo refinement following more empirical research.

Supplementary Methods

Study design, participants, and procedures

Participants ($N = 136$) aged 18-77 years ($M = 35.6$, $SD = 13.1$) formed a convenience sample, who responded to a study advert:

This study is about the compensation strategies used to fit in, when someone feels socially different to others around them. We are interested in the strategies, approaches and techniques that people may use to get by in social situations, when they feel that they aren't naturally 'getting it' socially. We would like to hear from anyone who has used strategies in this way, now or in the past. This may include, but is not restricted to, individuals on the Autism Spectrum.

Participants' information about sex and gender was requested with the choice of '(cis)male', '(cis)female', 'transgender male', 'transgender female' or 'please specify using the free-text response box'. This approach to recording sex and gender is in line with recent autism research.[4] All participants also reported information about any comorbid psychiatric diagnoses, their country of residence, employment status, and whether or not they lived independently. Diagnosed participants confirmed their diagnosis [Asperger Syndrome ($n = 33$), Autism Spectrum Disorder ($n = 19$), Atypical Autism ($n = 2$), Pervasive Developmental Disorder-Not Otherwise Specified ($n = 3$)] and gave details of the healthcare professional(s) who made the diagnosis. All Diagnosed participants, regardless of country of residence, had a diagnosis based on a version of the Diagnostic and Statistical Manual of Mental Disorders,[5] and/or the International Classification of Diseases.[6]

Participants then completed the 10-item Autism-Spectrum Quotient (AQ)[7] and responded to a number of open-ended questions (see below, p 4), which were designed to elicit information about compensatory strategies. Importantly, these questions were co-designed with three autistic adults to ensure questions were understandable and meaningful to autistic participants, in line with best practice in participatory autism research.[8] Participants were encouraged to give full answers and examples where possible. Finally, participants reported, using 5-point Likert scales, how successful and tiring their strategies were, and the likelihood of recommending them to others with social difficulties.

Supplementary Results

Additional quotations supporting the themes/subthemes and a comprehensive list of strategies are shown in Supplementary Tables 2 and 3, respectively. Group comparisons on theme endorsement, including effect sizes, are shown in Supplementary Table 4.

Participant demographics, comorbid diagnoses, and misdiagnoses were compared between groups (Main Text, Table 1). Additionally, the total number of comorbid diagnoses and misdiagnoses were

computed for each participant. There was no significant difference in comorbid diagnoses between groups (Diagnosed: $Md = 1$, $IQR = 2$; Self-identified: $Md = 1$, $IQR = 2$; Non-diagnosed: $Md = 0$, $IQR = 1$), $\chi^2(2) = 4.45$, $p = .11$. Groups differed on the total number of misdiagnoses (Diagnosed: $Md = 0$, $IQR = 1$, Self-identified: $Md = 0$, $IQR = 1$, Non-diagnosed: $Md = 0$, $IQR = 0$), $\chi^2(2) = 12.76$, $p = .002$, as they were greater in the Diagnosed and Self-identified than the Non-diagnosed group, $U = 1219.00$, $p = .001$, Bonferroni-corrected $p = .002$, $U = 396.50$, $p = .008$, Bonferroni-corrected $p = .024$, respectively. There was no significant difference between the Diagnosed and Self-identified groups, $U = 545.00$, $p = .94$, Bonferroni-corrected $p = .9\dot{9}$. AQ scores showed a comparable distribution to previous studies[7] and good internal consistency ($\alpha = 0.76$).

Supplementary References

1. Livingston LA, Happé F. Conceptualising compensation in neurodevelopmental disorders: Reflections from autism spectrum disorder. *Neurosci Biobehav Rev* 2017; **80**: 729–42. doi: 10.1016/j.neubiorev.2017.06.005
2. Lai M-C, Lombardo MV, Ruigrok ANV, et al. Quantifying and exploring camouflaging in men and women with autism. *Autism* 2017; **21**: 690–702. doi: 10.1177/1362361316671012
3. Hull L, Petrides KV, Allison C, et al. "Putting on my best normal": Social camouflaging in adults with autism spectrum conditions. *J Autism Dev Disord* 2017; **47**: 2519–34. doi: 10.1007/s10803-017-3166-5
4. Cooper K, Smith LGE, Russell AJ. Gender identity in autism: Sex differences in social affiliation with gender groups. *J Autism Dev Disord* 2018; **48**: 3995–4006. doi: 10.1007/s10803-018-3590-1.
5. American Psychiatric Association. Diagnostic and statistical manual of mental disorders. 5th ed. Washington, DC: American Psychiatric Publishing; 2013.
6. World Health Organization. The ICD-10 classification of mental and behavioural disorders: clinical descriptions and diagnostic guidelines. Geneva: World Health Organization; 1992.
7. Allison C, Auyeung B, Baron-Cohen S. Toward brief "red flags" for autism screening: The Short Autism Spectrum Quotient and the Short Quantitative Checklist for Autism in Toddlers in 1,000 cases and 3,000 controls. *J Am Acad Child Adolesc Psychiatry* 2012; **51**: 202–12. doi: 10.1016/j.jaac.2011.11.003
8. Fletcher-Watson S, Adams J, Brook K, et al. Making the future together: Shaping autism research through meaningful participation. *Autism* 2019; **23**: 943–53. doi: 10.1177/1362361318786721

Open-ended Questions

- What was the experience of gaining a diagnosis like for you? Were there any obstacles to getting diagnosed? **OR** When and how did you come to self-identify as autistic? **OR** When and how did you come to realise that you were socially different to other people?
- Have you ever received a diagnosis for a condition that you felt was inaccurate? If so, please describe.
- As a child or teenager, were there any strategies that you used in social situations to compensate for or make up for your social differences?
- Where did these strategies come from? For example, were you taught them, did you read about them, did you create them?
- As an adult now, are there any strategies that you use in social situations to compensate for your social differences?
- Where did these strategies come from? For example, were you taught them, did you read about them, did you create them?
- Are there any strategies that you use to work out what other people are thinking or feeling?
- Where did these strategies come from? For example, were you taught them, did you read about them, did you create them?
- How do you think you compare as an adult now to how you were as a child?
- Can you identify any key moments in your life (in childhood and/or adulthood) which prompted you to begin to use strategies in social situations?
- How do you know when your strategies are or are not working in a social situation?
- Have you ever or do you currently find social situations tiring?
- Are there any particular things, contexts or people that help you/make it difficult for you to use strategies in social situations?
- Do you alter your strategies depending on who you are interacting with?
- Which of your abilities (including any neurodiverse ways of thinking) do you rely upon to compensate in social situations?
- Are you always aware of when you are using strategies to compensate or have some become automatic?
- Would you say that you are different at home alone compared to when you are in social company?
- Do people ever tell you that you seem just like everyone else (i.e., doubt that you are socially different)?
- In your own words, how would you describe compensation and what does the experience feel like for you?
- Would you say that compensating in social situations helps or hinders (or both) with achieving your life goals?
- Would you say that compensating in social situations is desirable or not?
- Do you have (or have you had) any mental health difficulties (e.g., anxiety, depression)? If so, do you think your mental health difficulties are related to compensating?
- In your opinion, do neurotypical (i.e., socially 'typical') individuals use strategies in social situations? If so, how do you know?
- Which qualities do you think make someone socially skilled?
- How easily can you spot when other individuals who are socially different (i.e., people who also feel they are not socially 'getting it') are using strategies to compensate?

Supplementary Table 1. Hypothetical distinctions between mechanisms of Shallow Compensation, Deep Compensation, Genuine Remediation and Delayed Maturation. Social skills at the behavioural level and Theory of Mind (ToM – the ability to understand other minds) at the cognitive level are used as an example here, but could be substituted with another behaviour and underlying cognitive construct. Adapted from Livingston & Happé.[1]

	**Shallow Compensation**	**Deep Compensation**	**Genuine Remediation**	**Delayed Maturation**
Behavioural	Good social skills in structured contexts (e.g., ADOS assessment) but does not hold up in everyday situations where social cues are ambiguous and fast-paced	Good in structured contexts and everyday situations	Good in structured contexts and everyday situations	Good social skills in structured contexts but may not hold up in complex social situations, where e.g. multiple sources of information must be integrated.
Cognitive	Clear ToM deficit when measured with sensitive (e.g., implicit ToM) task. May be able to solve explicit ToM tasks through logical reasoning.	No clear ToM deficit (compensation has extended to cognitive level). Good ToM task performance might come at cost to time.	No ToM deficit on implicit or explicit ToM tasks. ToM deficit has genuinely resolved.	No clear ToM deficit but ToM may have matured too late to have dynamic input into the system. Might be able to attribute mental states, but not as fast, not integrating multiple cues, etc.
Neural	ASD-associated brain atypicalities persist; e.g. different patterns of brain activation during ToM tasks.	Atypical neural route that supports good cognitive task performance e.g., extra neural 'effort', recruitment of additional brain areas.	Unknown. Potentially a combination of typical neural markers and traces of early ASD markers.	Unknown. Could be lasting functional atypicalities; e.g., connectivity to other brain regions may be different, if critical windows missed for neural integration of different functions.
Genetic	Substantial genetic load for ASD as well as genetic propensity for general-purpose cognitive skills (e.g., IQ)	Substantial genetic load for ASD as well as genetic propensity for general-purpose cognitive skills (e.g., IQ)	Reduced genetic load for ASD compared to compensators.	Reduced genetic load for ASD compared to compensators.
Other Characteristics	Effortful, absorbs central resources, hence breaks down under stress/anxiety, and may come at a cost to mental health.	Potentially some cognitive/brain marker at some point in earlier development. May not negatively impact mental health.	No more effortful than for neuroptypicals, no difference on e.g. dual task challenges.	Longitudinal studies might show neurotypical order of skill acquisition, but with delay?

Supplementary Table 2. Additional example quotations for the eight themes and eighteen subthemes.

**Theme**	**Subtheme**	**Example Quotation**[a]
**A Secondary Route**	Cognitively Taxing	*I know the way to present myself and what is and what is not socially appropriate to say. However, I struggle in the regulation of my own behavior. I generally have impairments on things like inhibitory control tasks, and I wonder if this causes the dissociation.* (M S 25-30yo)
		Usually if I have to keep it up for a long time then the situation deteriorates. (F N 51-60yo)
	An Upper Limit	*It's an art, not a science. And it is only ever partial in implementation or approach* (M D 25-30yo)
		The biggest sign is when some is overcompensating by basically bad acting a role script, such as repeating a phrase, posture or gesture overly. (M S 41-50yo)
**Gap Between Appearance and Internal Reality**	Shallow Compensation	*It came to me in a flash that if I acted like 'that person over there' no-one would know that I was struggling inside…I made mental lists of the things I had to remember to say /do)* (F N 41-50yo)
		I am now able to identify a joke by intonation & body language (without necessarily knowing it's funny) (F D 31-40yo)
	Deep Compensation	*For me, it's easy to find patterns in systems or programs... I apply that same skill…to people and social situations.* (F N 18-24yo)
**Behavioural Masking**	-	*Usually I find it easier just to not talk at all in many situations than exhaust myself behaving correctly.* (F S 25-30yo)
		Pretending to be interested or enthusiastic in a social event is nothing strange for the typical person. Example: putting on a smile when your mother-in-law comes over for dinner (M N 31-40yo)
**Internal Factors**	Individual Differences	*I will remember what someone said about themselves, or about a subject, even if it was a long time ago. I notice small details about someone's attitudes or behaviours.* (F N 31-40yo)
	Social Motivation	*I really like people and I do my best therefore to understand them. I have always valued 'feedback' from others who have told me I might have been interrupting a bit…I don't like to think I am being unfair.* (F D 51-60yo)

	Costs Versus Benefits	*Sometimes, I can't be bothered. Plus, there is often smaller benefit for me as I do not need the friendship that much and the conversation is usually social in nature, which I just don't find interesting.* (F N 41-50yo)
**External Factors**	For Others	*When I had my children, I forced myself to go to mum and baby groups so I could meet other mums…I knew that if I didn't I would end up staying at home, and the children would not see other children.* (F D 31-40yo)
		With people I don't know I ramp up my strategies, monitor myself a lot and I will only talk about safe topics that I know they will like…with people in authority (such as a manager or someone in charge) I can't use my being quiet strategy so I have to really monitor myself and observe them very closely. (F S 25-30yo)
	Environmental Demands	*If there is too much sound, loud music or people talking over each other or bright lights, then I shutdown and cannot use my strategies.* (N D 31-40yo)
		I will have felt sort of normal for some time and then something will happen and I will realise that I am really not. (F N 25-30yo)
	Interaction Is Two-way	*I occasionally meet people who share my love of independent backpacking or politics/social sciences or nature or art, then we can talk happily for hours.* (F N 61-80yo)
**Diagnosis and Support**	Late Diagnosis	*It was suspected I was autistic from early on but because I was born a girl it was overlooked, I had coping strategies and was better able to blend in.* (TM D 25-30yo)
		All the symptoms they outlined at age 6 are all under the rubric of autism but it wasn't named autism because [I]…did well on the IQ tests and kept my head down. (F D 41-50yo)
	Looked Too Normal	*[Compensation] helped at work but…ultimately, I always ended up with problems in the workplace. It would have been nice to have had a diagnosis then because maybe people would have been kinder and more supportive*. (F D 41-50yo)
		I have on several occasions gone on "dates" where I did not realise the occasion was intended as a date until attempted sexual contact was made. (F N 31-40yo)
**Quality Of Life**	Health and Wellbeing	*I still have social anxiety - quite majorly - just because the risk is always there that I look unusual or say something embarrassing, or miss a social cue and make someone feel awkward or insulted.* (M D 18-24yo)
		The experience feels like you are running a marathon while non-autistics are casually walking. It is draining. It likely led to long bouts of depression before I knew I was autistic. It fuels my anxiety and hinders my everyday functioning. (F D 31-40yo)

		[Compensation is] necessary, in the same way that washing yourself is necessary…Without maintaining some minimal acceptable level of social behaviour…you risk being pushed outside that community…Unfortunately, most of us will need that community fairly often. John Donne had the right of it about no man being an island. (F D 25-30yo)
	A Role In Society	*It is a vital skill that allows me to survive in a neurotypical world. While some people embrace my otherness, it is often not accepted in professional environments.* (F D 25-30yo)
		Compensating prepared me for parenthood because I am used to taking on a role and not expecting things to really be about me. (F D 41-50yo)
	Self and Social Relationships	*It helped to get me out there and be able to meet a partner because I'd much rather choose to stay home if I could.* (F N 41-50yo)
		When my children were younger I often felt exploited as I was very often the person to offer hospitality to other parents and children…and would be very generous with time, money and resources to others in ways I felt were rarely reciprocated. (F S 51-60yo)
		Compensation…is denying your authentic self in order to squeeze yourself into a mould the world thinks you ought to fit…it's always other-focused and so can cause conflict with your true self-concept (F S 25-30yo)
**Trajectories and Attitudes**	Things Are Better Now	*People generally perceive me as interesting or eccentric now, rather than being repulsed or a source of mockery… as a teenager most people would have agreed that there was something "off".* (TF D 41-50yo)
	Balance Is Key	*The healthy way to compensate is to make sure there is also a pressure-release valve in the form of down time and me time/special interest time or time with autistic friends.* (F D 41-50yo)
		I wish I knew then what I know now, and would have not tried as hard to fit in. (F N 25-30yo)
	An Ongoing Challenge	*I would have loved to have a proper career, but I can't even pass as normal in a menial job, so it is not realistic to think I could succeed at a higher level.* (M S 41-50yo)
		When I was a younger adult, and especially still in higher education, difference was less noticeable. Now, when most of my peers are in successful careers and relationships and living independently, my difference stands out more than ever. (M D 41-50yo)

[a] Each example quotation is accompanied by text in parentheses indicating the participant's gender (F = female, M = male, TF = transgender female, TM = transgender male, N = nonbinary), group status (D = Diagnosed, S = Self-identified, N = Non-diagnosed) and age range.

Supplementary Table 3. Description of strategies (Behavioural Masking, Shallow Compensation, Deep Compensation, Accommodation).

Strategy Type	Description
**Behavioural Masking**<br>Strategies that involve regulating (increasing/dampening) pre-existing social behaviours. They 'hide' autistic characteristics superficially from a far, but do not support active participation in two-way interaction.	
Avoidance	Avoid social situations where you would stand out.
Hold back	Hold back your true thoughts and opinions in conversation (e.g., agree with others even if you disagree with them, tolerate behaviour of others). Hide aspects of your personality that would be deemed different to the norm (e.g., your interests and hobbies).
Suppress	Suppress atypical behaviours (e.g., hand flapping, fidgeting).
Present but passive	Attend social events, even if you would rather not, to give the impression of sociability. Stand in a conversation but say/do as little as possible.
Superficial assimilation	Dress and speak like the group you are trying to blend in with (e.g., copy hairstyle, language, interests).
Basic social etiquette	Reflect basic social etiquette to indicate a willingness to socialise (e.g., smile, manners, look towards other people).
**Shallow Compensation**<br>Strategies that involve actively producing new social behaviour that otherwise does not come naturally. They support participation in two-way interaction, without supporting underlying social cognition, meaning they are inflexible and not always effective.	
Plan and rehearse	Predict, plan out and rehearse conversations before they happen, out loud or in your head.
Copied behaviour	Mimic phrases, gestures, facial expressions, tone of voice picked up from other people and/or TV/film/book characters.
Eye contact	Make appropriate eye contact, even if it is not useful for communication and/or is aversive. Avoid eye contact but give the impression of social interest (e.g., look at bridge of nose, stand at a 90° angle to interaction partner).
Learned scripts/social rules	Enact learned scripts and social rules, even when it may not be appropriate, to guide conversations (e.g., ask others set questions, small talk, laugh at 'joke cues', turn-take in conversation).

Guide conversation	Steer conversation to topics you are equipped to talk about (e.g., special interests). Focus conversation on your interaction partner to draw attention away from self.
Rely on others	Attend social events with a more socially-skilled individual who can act as a 'social crutch' (e.g., introduce you, fill in or disguise your social mistakes, explain social nuances to you).
Reduce social demands	Reduce social demands on yourself in order to disguise any social faux pas (e.g., 'flit' between different groups/conversations, engage in 1:1 conversation rather than groups so there are less social signals to read, make use of structured socialising or 'organised fun').
Counselling skills	Listen to, repeat and rephrase what your interaction partner says to give the impression of being an 'good listener' or 'adviser', without having to necessarily mentalise.
Use props	Rely on props (e.g., dog, children, interesting object) to structure and guide conversation. Similar to learned scripts.
Play a false role	Play an exaggerated role or character that is inconsistent with the 'real you' (e.g., false confidence, fabricated stories, extraverted personality).
**Deep Compensation**<br>Strategies that enable an alternative route to social cognition, for example, to mentalise or 'solve' theory of mind. They support participation in two-way interaction. They are more flexible, less prone to breakdown and ultimately more effective than shallow strategies.	
Learned non-verbal cues	Use learned rules about non-verbal behaviour (e.g., facial expression, body language, direction of gaze), when it is appropriate, to infer what others are thinking/feeling. For example, inferring that when someone looks at the ground or rolls their eyes, they are bored.
Learned verbal cues	Use learned rules about verbal behaviour (e.g., tone of voice, content of speech) to infer what others are thinking/feeling. For example, inferring that someone who is talking about a funeral with a particular tone is likely sad.
Assess behaviour	Assess someone's behaviour over time to infer what they are thinking/feeling. For example, if someone re-invites you to a social event, they think positively of you.
Substituted perspective taking	Substitute someone else's values/preferences/interests with your own or those of a TV/film/book character to infer what others are thinking/feeling. For example, if someone is acting similar to a TV/film/book character in particular situation, infer that they are thinking/feeling how that character would in the same situation.
Logic/context/experience	Predict likelihood of what someone is thinking/feeling based on logic, the context or experience of how that person has previously behaved. Often involves analysing social situations after they have happened and carrying 'lessons learned' to the next time the same situation happens.
Flexible catalogue	Flexibly use built catalogue of possible interpretation of others' thoughts/feelings, based on combination of multiple sources of information (e.g., logic, context, facial expression, tone of voice).

More information/time	Gain more information to increase accuracy of your inference about someone's thoughts/feelings (e.g., get them to repeat what they have said in a different way, find out about their interests/opinions from others). Gain more time to make a judgement of someone's thoughts/feelings (e.g., take a well-timed break to consider various interpretations).
Recalibrate	Recalibrate your interpretation of someone else's thoughts/feelings based on self-awareness of your own cognitive biases (e.g., tendency to perceive neutral expression as anger).
Psychological theory	Apply learned psychological theory to help infer what others are thinking/feeling (e.g., categorise people by personality type).
**Accommodation Strategies**<br>Strategies that help accommodate, but do not necessarily alter, your social differences	
Play to your strengths	Play to your strengths (e.g., humour, wit, intelligence) to add additional value to conversation with others, despite your social differences.
Be helpful/liked	Go out of your way to be helpful to others, so that your social differences might be forgiven.
Seek 'atypical' others	Seek relationships with others who are also socially 'atypical' and therefore more accepting of your social differences.
Accommodating employment	Work in an environment where your social differences are actively accommodated (e.g., 'autism friendly' workplace) or where non-social skills are valued over social ones (e.g., academia, skill-based job).
Foreign disguise	Live in a foreign country so that your differences are attributed to being foreign by others. Live in your country of birth but seek relationships with others who are foreign, so that your social differences are attributed to cultural differences.
Disclose difficulties	Disclose your difficulties or diagnosis to others so that they can better accommodate you.

Supplementary Table 4. Number of participants endorsing each theme or subtheme, by gender, group and autistic behaviours.

	**Gender***			**Group**				**Autistic Behaviours†**		
	Male (n = 29)	Female (n = 97)	p, Φ	Diagnosed (n = 58)	Self-identified (n = 19)	Non-diagnosed (n = 59)	p, Φ	High (n = 89)	Low (n = 47)	p, Φ
**A Secondary Route**										
Cognitively Taxing	26(90)	92(95)	.38, 0.09	55(95)	18(95)	55(93)	.99̇, 0.03	83(93)	45(96)	.71, -0.05
An Upper Limit	22(76)	59(61)	.19, -0.13	40(69)	13(68)	35(59)	.53, 0.10	61(69)	27(57)	.26, 0.11
**Gap Between Appearance and Internal Reality**										
Shallow Compensation	26(90)	93(96)	.35, 0.11	55(95)	17(89)	57(97)	.42, 0.11	88(99)‡	41(87)‡	.007, 0.25
Deep Compensation	27(93)	96(99)	.13, 0.16	57(98)	19(100)	57(97)	.99̇, 0.08	88(99)	45(96)	.55, 0.10
**Behavioural Masking**	29(100)	96(99)	.99̇, -0.05	57(98)	19(100)	59(100)	.57, 0.10	88(99)	47(100)	.99̇, -0.06
**Internal Factors**										
Individual Differences	29(100)	96(99)	.99̇, -0.5	57(98)	19(100)	59(100)	.57, 0.10	88(99)	47(100)	.99̇, -0.06
Social Motivation	24(83)	91(94)	.13, 0.17	50(86)	19(100)	56(95)	.094, 0.19	81(91)	44(94)	.75, -0.05
Costs Versus Benefits	20(69)	75(77)	.46, 0.08	38(66)	16(84)	46(78)	.15, 0.16	64(72)	36(77)	.68, -0.05
**External Factors**										
For Others	26(90)	95(98)	.080, 0.18	56(97)	17(89)	58(98)	.17, 0.15	88(99)§	43(91)§	.048, 0.19
Environmental Demands	28(97)	90(93)	.68, -0.07	55(95)	18(95)	55(93)	.99̇, 0.03	85(96)	43(91)	.45, 0.08
Interaction Is Two-way	27(93)	96(99)	.13, 0.16	56(97)	18(95)	59(100)	.28, 0.14	88(99)	45(96)	.55, 0.10
**Diagnosis and Support**										
Late Diagnosis	24(83)	87(90)	.33, 0.09	52(90)	18(95)	50(85)	.48, 0.11	83(93)§	37(79)§	.022, 0.22
Looked Too Normal	21(72)	77(79)	.45, 0.07	47(81)	15(79)	43(73)	.57, 0.09	70(79)	35(74)	.67, 0.05
**Quality Of Life**										
Health and Wellbeing	27(93)	96(99)	.13, 0.16	55(95)	18(95)	59(100)	.20, 0.15	86(97)	46(98)	.99̇, -0.04
A Role In Society	17(59)	62(64)	.66, 0.5	40(69)	11(58)	37(63)	.63, 0.08	56(63)	32(68)	.58, -0.05
Self and Social Relationships	25(86)	83(86)	.99̇, -0.01	48(83)	16(84)	53(90)	.56, 0.10	78(88)	39(83)	.60, 0.06
**Trajectories and Attitudes**										

Things Are Better Now	19(66)	67(69)	.82, 0.03	34(59)§	13(68)§	47(80)§	.047, 0.21	57(64)	37(79)	.084, -0.15
Balance Is Key	24(83)	86(89)	.52, 0.08	50(86)	18(95)	50(85)	.59, 0.10	79(89)	39(83)	.43, 0.08
An Ongoing Challenge	16(55)	39(40)	.20, -0.13	23(40)	9(47)	27(46)	.77, 0.07	42(47)	17(36)	.28, 0.11

Data are number (%) of participants giving at least one example fitting each theme/subtheme. Each participant received a maximum of one count per theme/subtheme. Fisher's exact tests compared frequencies across group, gender, and autistic behaviours. Φ=0.1 indicates a small effect size, Φ=0.3 indicates a medium effect size, and Φ=0.5 indicates a large effect size.

*Participants reporting other gender were not included in these analyses. †Collapsing across diagnostic groups, participants were split into high and low autism-spectrum quotient score groups: the high group had a score of 6 or more, in line with the clinically significant cutoff[7] and the low group had a score of less than 6. ‡p<0·01. §p<0·05.
